# Complete Genome Sequences of Six Salmonella enterica Serovar 4,[5],12:i:– Isolates from Canada

**DOI:** 10.1128/MRA.00074-20

**Published:** 2020-06-11

**Authors:** Matthew Walker, Chrystal Landgraff, Clifford Clark

**Affiliations:** a Division of Enteric Diseases, National Microbiology Laboratory, Public Health Agency of Canada, Winnipeg, MB, Canada; Portland State University

## Abstract

Salmonella enterica with antigenic formula 4,[5],12:i:– is a monophasic variant of S. enterica serovar Typhimurium that has emerged globally as a human pathogen over the last 3 decades. We describe the closed genomes and plasmids from six S. enterica 4,[5],12:i:– isolates recovered from stool samples obtained during investigation of human food poisoning cases reported to PulseNet Canada that are suitable for use as reference strains.

## ANNOUNCEMENT

We present here the complete genome sequences of six Canadian Salmonella enterica subsp. *enterica* serovar 4,[5],12:i:– isolates. Bacteria were isolated from patient stool samples by commercial laboratories, sent to Canadian provincial public health laboratories for further characterization, and provided to the National Microbiology Laboratory (NML) for whole-genome sequencing. Strains were grown and maintained on nutrient agar plus 0.5% NaCl at 37°C and stored in 19% skim milk. *Salmonell*a serotyping was performed at the NML following conventional agglutination methods. Genomic DNA was isolated from overnight cultures grown at 37°C on nutrient agar using the Epicentre metagenomic DNA isolation kit for water following the manufacturer’s guidelines. Paired-end sequencing (2 × 300 bp) of Nextera XT genomic DNA libraries was performed on an Illumina MiSeq sequencing platform using the MiSeq reagent v3 kit and 600 cycles, yielding 58× to 139× genome coverage (Table 1).

**TABLE 1:** NCBI accession numbers and summarized sequencing and genome characteristics of *Salmonella* 1,4,[5],12:i:– isolates sequenced on Illumina MiSeq and Oxford Nanopore MinION sequencing platforms^*a*^

Isolate name	SRA accession no. (MiSeq)	SRA accession no. (ONT)	GenBank accession no.	No. of reads (MiSeq)	No. of reads (ONT)	MiSeq genome coverage (×)	ONT genome coverage (×)	Genome size (bp)	GC content (%)
PNCS009777	SRR11194321	NA	CP036174.1	1,050,482	NA	115	NA	4,981,102	52
PNCS014854	SRR11194316	SRR11215570	CP037874.1	991,949	7,393	60	14.58	4,817,234	52
PNCS015054	SRR11194318	SRR11215569	CP037877.1	1,080,261	7,237	106	12.31	4,786,287	51
PNCS014863	SRR11194320	SRR11215568	CP037879.1	957,395	28,928	58	77.41	4,984,391	52
PNCS009991	SRR11194319	NA	CP037881.1	2,433,867	NA	139	NA	5,036,850	52
PNCS014875	SRR11194317	SRR11215567	CP037882.1	1,009,327	14,833	61	39.47	4,996,832	52
pPNCS009777_S1	SRR11194321	NA	CP036173.1	NA	NA	NA	NA	4,418	53
pPNCS014854_S1	SRR11194316	NA	CP037873.1	NA	NA	NA	NA	93,844	53
pPNCS015054_S2	SRR11194318	NA	CP037875.1	NA	NA	NA	NA	247,701	53
pPNCS015054_S3	SRR11194318	NA	CP037876.1	NA	NA	NA	NA	96,545	53
pPNCS014863_S1	SRR11194320	NA	CP037878.1	NA	NA	NA	NA	6,760	46
pPNCS009991_S1	SRR11194319	NA	CP037880.1	NA	NA	NA	NA	40,085	41

a Isolates were not closed using MinION sequencing; plasmid sequencing data are included with the full-genome sequences. NA, not applicable.

The genome sequences for strains PNCS009777 and PNCS009991 (GenBank accession numbers CP036174 and CP037881, respectively) were closed and finished as previously reported (1). Oxford Nanopore Technology (ONT) MinION sequencing technology was employed to produce long reads for PNCS014854, PNCS015054, PNCS014863, and PNCS014875, enabling genome closure. ONT libraries were prepared using the one-dimensional (1D) sequencing kit (SQK-LSK108) with a native barcoding kit (EXP-NBD103) and an R9.4 flow cell (FLO-MIN106). Sequencing was performed for 24 h, yielding a total of 0.91 Gb of long-read sequencing data. Hybrid assemblies were produced using Unicycler v0.4.3 with error correction (2). The remaining contigs were closed and finished using Staden gap v4.10 (3), and a combination of PCR and Sanger sequencing was used for gap closure. These methods were run using default settings to generate closed, finished genomes consisting of a single chromosome and one or more plasmids.

Prior to annotation using the NCBI Prokaryotic Genome Annotation Pipeline (PGAP) (4), genome sequences were trimmed and oriented to the *dnaA* gene of S. enterica serovar Typhimurium LT2. During the process of genome closure, plasmids were identified as nonchromosomal contigs with overlapping ends and were circularized using methods reported previously (1). With the exception of strain PNCS014875 (GenBank accession number AAQBXA000000000), each isolate harbored at least one plasmid and one strain, and strain PNCS015054 (AAQBXH000000000) was found to carry two plasmids (Table 1). Plasmid sequences were compared to sequences deposited in NCBI by performing standard nucleotide BLASTn searches against the standard nonredundant (nr) databases (5). Plasmids pPNCS015054_S3 (NZ_CP037876) and pPNCS014854_S1 (NZ_CP037873) displayed 99.98% identity with 97% coverage and 99% identity with 100% coverage, respectively, with an unnamed plasmid from *S.* Typhimurium E40V (CP038435). Plasmid pPNCS015054_S2 (NZ_CP037875) displayed the highest genetic identity to the S. enterica strain F8475 plasmid pF8475 (KP899804) but carried an additional ∼36.4 kb of DNA. Plasmid pPNCS009991_S1 (NZ_CP037880) was 99.96% identical (100% coverage) to IncX plasmid pYU39 (CP011431.1) and 99.98% identical (100% coverage) to pExPB5-59-1 (MF428416). Plasmid pPNCS009777_S1 (NZ_CP036173) shared identity to E. coli plasmids PCN061p2 (CP006638; 95.65% identity, 84% coverage) and pRCS49_pIII (LT985254; 95.10% identity, 84% coverage). Plasmid pPNCS014863_S1 (CP037878) was 98.83% identical (100% coverage) to S. enterica serovar Newport plasmid pSNE1-1926 (CP025235).

These sequences provide high-quality references for genome assemblies of *Salmonella* 4,[5],12:i:– and for future virulence analyses or population structure studies of this clinically relevant monophasic S. enterica serovar.

### Data availability.

The complete genome sequences were deposited under the NCBI BioProject number PRJNA522746 and the GenBank database under the following accession numbers: CP036174.1 (PNCS009777), CP036173.1 (pPNCS009777_S1), CP037874.1 (PNCS014854), CP037873.1 (pPNCS014854_S1), CP037877.1 (PNCS015054), CP037875.1 (pPNCS015054_S2), CP037876.1 (pPNCS015054_S3), CP037879.1 (PNCS014863), CP037878.1 (pPNCS014863_S1), CP037881.1 (PNCS009991), CP037880.1 (pPNCS009991_S1), and CP037882.1 (PNCS014875). The MiSeq and ONT accession numbers and basic genome statistics are summarized in Table 1.
